# The Effect of Treatment during A Haze/Post-Haze Year on Subsequent Respiratory Morbidity Status among Successful Treatment Tuberculosis Cases

**DOI:** 10.3390/ijerph16234669

**Published:** 2019-11-23

**Authors:** Suyanto Suyanto, Alan Geater, Virasakdi Chongsuvivatwong

**Affiliations:** 1 Epidemiology Unit, Faculty of Medicine, Prince of Songkla University, Hat Yai 90110, Thailand; alan.g@psu.ac.th (A.G.); cvirasak@medicine.psu.ac.th (V.C.); 2 Department of Public Health, Faculty of Medicine, Riau University, Pekanbaru 28133, Indonesia

**Keywords:** TB, air pollution, respiratory morbidity, year of treatment

## Abstract

The purpose of this study was to evaluate the respiratory morbidity status within the two to three years among successful (completed/cured) treatment of tuberculosis cases during a haze year (2015) and a post-haze year (2016). The study was conducted among 133 cases of a 2015 group and 103 cases of a 2016 group between January to March 2018 in Pekanbaru city, Indonesia. The St George Respiratory Questionnaire (SGRQ) was used to assess respiratory morbidity status. A higher score corresponds to worse respiratory morbidity. Based on a directed acyclic graph, quantile regression models were constructed to assess the associations between haze/post-haze year and the SGRQ (symptom, activity, impact, and total) domains score. The subsequent respiratory morbidity status of tuberculosis (TB) cases was poorer among respondents treated during a haze year (2015). Among SGRQ domains, only the activity domain score showed significant difference, in which the median for the 2015 group was 23.7 (inter-quartile range (IQR); 17.2, 30.9) compared to 18.4 (IQR; 11.9, 24.8) for the 2016 group. The effect was limited to the 2015 group who were exposed by an average PM_10_ index ≥ 55 during TB treatment. This raises concern for monitoring and improving the quality of life of TB patients treated during a haze year.

## 1. Introduction

Tuberculosis (TB) is one kind of chronic respiratory disease still posing a public health challenge in Indonesia [1]. The morbidity of this disease includes not only physical symptoms (cough and difficult breathing) but also psycho-social aspects (worry, lack of energy, and feeling of depression), that need to be identified among patients. Both respiratory morbidity aspects are covered in Health-related Quality of Life (HRQoL) [2,3]. Despite successful TB treatment bringing significant benefit to patients in terms of relief from physical symptoms [4,5,6], their quality of life compared with the general population is still poor [6,7]. Several factors are already known to be associated with the quality of life of TB patients, including demographic (age, gender), socioeconomic (income, education, social security), behavior (smoking), therapy related (treatment adherence), and psycho-social aspects (stigmatization) [8,9,10].

Air pollution is the main environmental health problem in many countries in Asia [11,12,13]. The sources of ambient air pollution are mainly industrial emissions and pollution from burning of fossil fuel used for transportation. In addition, in some areas of Indonesia, the air pollution sources also from open land burning fire [14,15]. Following WHO air quality guidelines [13], governments in Asia have set efforts to control the ambient air quality level. In China, for example, the air pollution control policy has seen successfully improving air quality levels (PM_2.5_ indicator) in Yangtze River Delta cities [16]. Air quality index (AQI) standards have been adopted by the Indonesia government to protect public health from the negative effects of environmental pollutants and is known as the Index Standard Pencemaran Udara (ISPU) [17]. Indonesia air quality index (AQI) uses the level of PM_10_ for corresponding with the health advisory. However, Indonesia does not have a PM_2.5_ standard for its air quality level [18].

Air pollution exposure is associated with an increase of TB incidence in the short and long-term [19]. Studies in North Carolina [20] and Chengdu [21] reported that there is significant association between ambient PM_10_ and TB incidence. Moreover, after diagnosis has been established, a study in Taiwan that had subjects with TB positive culture annually exposed to ≥50 mg/m^3^ PM_10_ had an increased time required for sputum culture conversion [22]. Inhalation exposure of PM_10_ can aggravate airway disease by activating inflammatory reactions through increased mucus secretion in the long-term [23]. Further, it can increase susceptibility to respiratory symptoms [24], causing shortness of breath and exacerbation of symptoms of people with asthma and chronic obstructive pulmonary disease. 

Throughout July and October 2015, a haze of air pollution from forest fires occurred at high levels of air pollution in Sumatera Island, Indonesia [15]. Those pollutants can also be transported through air to other places, deteriorating the air in several Southeast Asia countries, such as Malaysia, Singapore, and southern Thailand [25]. However, there is a paucity of information on whether treatment in the year with haze has any effect on the subsequent respiratory morbidity of TB patients in Indonesia. Hence, the purpose of this study was to evaluate the respiratory morbidity status within the two to three years among successful (completed/cured) treatment of tuberculosis cases during a haze year (2015) and a post-haze year (2016) in Pekanbaru, Indonesia. 

## 2. Materials and Methods

### 2.1. Study Design and Setting

A cross-sectional study was conducted at public health centers (Puskesmas) in Pekanbaru District, Riau Province, Sumatera Island, Indonesia, between January to March 2018. This district was chosen because it was the worst affected by the haze in 2015. 

### 2.2. Study Population

**Figure 1:**
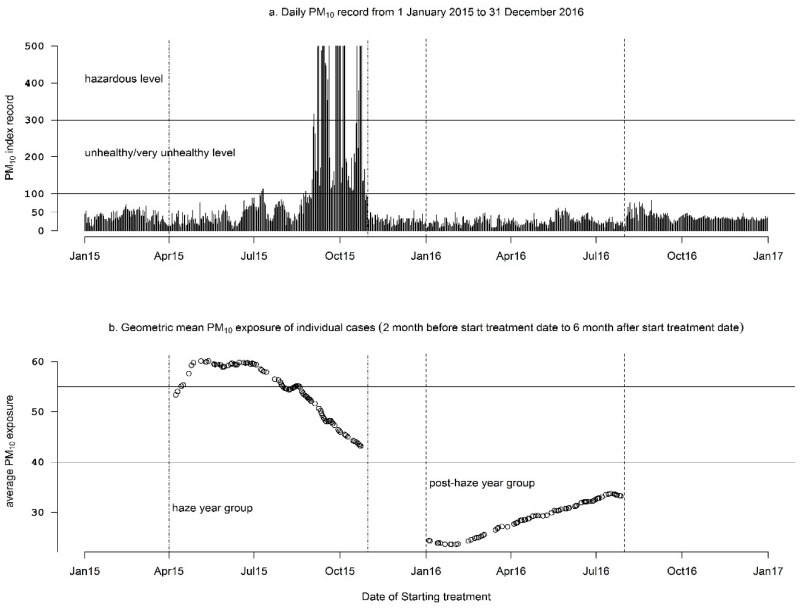
PM_10_ Air Quality Index record (**a**) and average PM_10_ exposure among respondents (**b**) according to the date of starting treatment.

The list of TB patients treated in the years 2015 and 2016 was extracted from the TB surveillance report. The TB study population frame was developed on two groups based on the pattern of the daily PM_10_ index. Information related to air quality data was obtained from the citywide Pekanbaru Environment report. Since the high air pollution levels were recorded between July to October 2015 (Figure 1a), patients who started treatment between 1st April and 30th October 2015 (haze-year group) were exposed to higher average air pollution level than those who started treatment between 1st January to 31st July 2016 (post-haze year group).

Inclusion criteria for the sampling frame were: confirmed new pulmonary TB case (with smear positive or smear negative on initial examination), having successful TB treatment (completed or cured), and aged between 18 and 70 years at the start of treatment. From this population frame, TB personnel in each public health center (Puskesmas) traced the cases’ medical records to ensure they had a telephone number and could be reached. Cases who were known to have already died, to have migrated, or to have any current severe cardiovascular diseases (Chronic Obstructive Pulmonary Disease (COPD), bronchitis, coronary heart disease), or to be pregnant according to the medical record were excluded. 

The required sample size was calculated based on detecting a difference between the means of two groups. The minimum clinically important difference in SGRQ scores has been reported to be four points [26]. The minimum sample size, assuming equal group sizes, for detecting a true difference in means between the exposure group and the comparison group of 4 points with a standard deviation of 10 points, was 99 per group. Using a simple convenience technique, about 150 respondents from each of the 2015 and 2016 groups were initially selected. About 20–30 cases per month of starting treatment of each group were contacted by telephone by the TB manager of each Puskesmas to explain the purpose of the study and invite them to the Puskesmas on a specified date to participate in the study.

### 2.3. Study Procedure

Data collection was done during the respondents’ visit to the Puskesmas. After receiving an explanation about the research objective, including the risks and benefits, respondents were asked to fill out a self-administered questionnaire. The research assistants then accompanied respondents to ensure they had answered all of the questions. Approximately 30–45 min was required to complete the questionnaire. The questionnaire collected information covering two areas:

A. Individual characteristics; age, sex, education, occupation, income, and current smoking behavior.

B. Respiratory morbidity status by using the Indonesian version of the St George’s Respiratory Questionnaire (SGRQ). This questionnaire was created by Professor Jones in the UK and is available online in the English language version [27].

Age was defined as the completed years of age at the time of data collection and categorized into young (<33 years), middle (33–49 years), and older (>49 years) age groups. Education group was defined by the highest formal educational level achieved and categorized into: low (primary and secondary education) and high (tertiary and university education). Occupation group was defined by the type of occupation performed daily and categorized into: outdoor job (informal worker, driver, parking officer, farmer) and indoor job (formal worker, household work, shopkeeper, student). Income group was calculated from the whole household monthly income divided by the number of household members and categorized into less than the median and more than or equal to the median. The smoking group was defined as those who currently used tobacco daily and categorized into smoker and non-smoker. 

For each participant, the medical record was retrieved to extract information, such as the date of starting treatment and the date of finishing treatment, initial Acid Fast Bacillus (AFB) sputum examination result, the month of negative sputum conversion and treatment result status. The initial AFB sputum examination result group was categorized as positive or negative. The conversion month group was defined as the month showing a negative smear result after the intensive phase treatment. The treatment result group was defined as the successful status at the end of treatment and classified as completed or cured. 

Daily citywide air quality data were gathered from Pekanbaru Environment Agency as the air quality index (AQI). AQI is an unitless number that is generated from the conversion of different ranges of air pollutant concentration measurement by an equation to indicate the level of danger to health. These AQI encompass the routinely measured pollutants, includes solid particulate (PM_10_), carbon monoxide (CO), nitrogen dioxide (NO_2_), ozone (O_3_), and sulfur dioxide (SO_2_). For each pollutant, a sub-index was calculated from a segmented linear function that transforms ambient concentrations into a scale extending from 0 through 500, which corresponds with a health advisory to the public [17]. The Indonesian classification of Air Quality Index (AQI) level [17] classified air quality into good (≤50), moderate (>50–100), unhealthy (>100–200), very unhealthy (>200–300), and hazardous (>300) level. 

Among major air pollutants, PM_10_ had received much attention [28]. Since the air quality in the immediate vicinity of each patient was not available, and the level of PM_10_ changed every day during the treatment period of each patient, the estimated average PM_10_ to which each case was exposed during treatment was calculated for each respondent as the geometric mean of the daily citywide mean PM_10_ air quality index from two months before the date of starting treatment until the last date of the 6-month treatment period. The level of average PM_10_ index exposure was categorized into 3 groups. These were determined based on the maximum geometric mean PM_10_ index of exposure among the 2016 group as reference (< 40) and the median of the PM_10_ index of 55 among the 2015 group.

### 2.4. Statistical Analysis

The SGRQ measures respiratory morbidity in 3 domains to give symptom (severity and frequency), activity (current state impairment of breathlessness), impact (social function and psychological disturbances), and total domain (overall health condition) scores. Each domain score and the total score are calculated from a weighted sum of the individual items in each domain. The scores can be obtained with the Excel-based SGRQ calculator, which can be downloaded from: www.readaptsante.com › 10-st-george_respiratory_questionnaire_calculator. The scales range from zero for optimal health to one-hundred for worst health [27].

Data were summarized as median with interquartile-range for SGRQ scores and number and percentage for each categorical variable. Comparisons between year and across groups were tested with the rank-sum test for SGRQ score data and chi-square for categorical data. A directed acyclic graph (DAG) was constructed to visualize the hypothesized association between variables of interest and identify the minimal sufficient adjustment set required for estimating the total effect of an exposure on the outcome [29]. Quantile regression models were constructed to identify significant predictors of the median SGRQ score. A *p* value < 0.05 was considered statistically significant in all analyses. 

### 2.5. Ethical Approval

Ethical approval (Reference number 60-330-18-2) was obtained by the Ethics Review Committee of Prince of Songkla University, Thailand.

## 3. Results

### 3.1. Respondents’ Characteristics

A total of 265 respondents attended the Puskesmas. However, only 236 respondents were enrolled. The remaining were excluded from this study for several reasons; 12 could not read or respond to the questionnaires well, eight refused to enroll, and nine were sick and visited Puskesmas for treatment. There were 133 cases from the 2015 group and 103 cases from the 2016 group who participated in the study. Statistically significant difference was found between cases treated in years 2015 and 2016 in term of PM_10_ exposure group (Table 1).

### 3.2. Air Pollutant Exposure 

Figure 1a displays the record of the daily average PM_10_ index in 2015–2016. The PM_10_ index started increasing above unhealthy levels (PM_10_ AQI > 100; PM_10_ > 150 µg/m^3^) at the end of July and reached the hazardous level (PM_10_ AQI > 300; PM_10_ 420 µg/m^3^) between September and October 2015. The warning of the hazardous level was declared by the government when the AQI reading reached a level of >300 on 9th September 2015. This status was abandoned when the AQI reading dropped to less than 200 at the end of October 2015. However, throughout 2016, the air quality remained in the good and moderate levels (<100).

Figure 1b summarizes the geometric mean of individual participant’s exposure to PM_10_ in the period from two months prior to and during the treatment period. Each point in Figure 1b indicates the geometric mean of the PM_10_ Index exposure plotted against the date of the starting treatment of each participant. Since the high air pollution levels were recorded between July to October 2015 (Figure 1a), patients who started treatment between 1st April and 30th October 2015 (haze year group) were exposed to a higher air pollution level than those who started treatment between 1st January to 31st July 2016 (post-haze year group).

### 3.3. Comparison of Respiratory Morbidity Status between Respondents Treated in 2015 and 2016

Table 2 shows the median total score and the scores of the three SGRQ domains (symptom, activity, and impact). The haze year group reported a significantly higher median score for activity domain than the post-haze year group.

### 3.4. Factors Associated with Score of SGRQ Score

**Figure 2:**
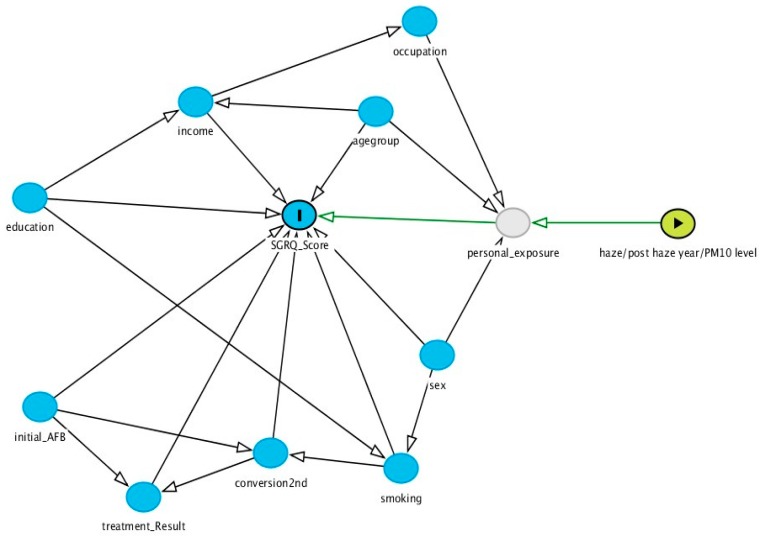
Example of a directed acyclic graph used to visualize causal pathways between haze/post-haze year and the SGRQ score.

Based on the pre-compiled DAG (Figure 2), quantile regression models were constructed to identify the associations between the haze/post-haze year and other potential predictor factors and the SGRQ domain score. Adults aged > 49 years and 33–49 years had, respectively, a 5.01 (95% CI 2.04, 6.52) and 9.49 (6.86, 11.08) points higher total domain score compared to those aged < 33 years, and the effect of age was significant in all domains. Males had significantly higher activity and total domain scores (Table 3). Respondents who reported being current smokers showed a 5.43 (1.19, 8.17) points higher total domain score than those who were non-smokers. This significant effect was seen in all SGRQ domains. Although, overall, the 2015 group had a significantly poorer activity domain score, subdividing the 2015 group based on the level of the PM_10_ index exposure revealed that those receiving an average PM_10_ index ≥ 55 in 2015 had a poorer outcome than the 2016 group.

## 4. Discussion

Identifying and improving the quality of life [2,3] is an important component in TB post-treatment management in the aspect of respiratory morbidity. Our findings show that, overall, the respiratory morbidity in TB cases was still poor within two-three years after their being declared cured or treatment completed. The subsequent respiratory morbidity of TB cases in the activity domain was significantly poorer among respondents treated during a haze year, but the effect was limited to those with an average PM_10_ index exposure ≥ 55. Smoking and older age were significantly associated with poorer respiratory morbidity in all SGRQ domains. 

There is no routine information available in Indonesia about respiratory morbidity assessment in TB treatment process [4,5]. In this study, we assessed the respiratory morbidity using St Georges Respiratory Questionnaires (SGRQ), which is a validated measurement tool for HRQoL for several chronic respiratory diseases [9,27], including in TB studies in the USA [30] and in Indonesia [31]. The SGRQ measures patients’ perception of respiratory problems in the past one to two years (symptom domain), disturbances to daily physical activity on the current date (activity domain), and disturbances of psycho-social function on the current date (impact domain) [9,27]. In our study, the overall SGRQ domain score in both groups was still higher (worse morbidity) than that of healthy subjects without a history of respiratory disease [9]. Our findings are consistent with previous studies, indicating that physical (symptom and activity domain) and psycho-social (impact domain) scores after successful treatment of respiratory diseases show a poorer score than in the general population [4,30,32,33]. 

Our study indicated that the overall of SGRQ score among patients treated in the haze year appeared higher than patients treated in the post-haze year. There was evidence that treatment during a haze year was significantly associated with worse morbidity in the activity domain. However, the effect was limited to those who were exposed by an average PM_10_ index ≥ 55 during TB treatment. Physiologically, PM_10_ can induce cough or breathlessness symptoms in the long-term [23]. Further, this may affect the respondent’s current perception of the activity domain question. Caution should be noted, however, as a high activity score may be related to persisting residual respiratory symptoms due to bronchial and parenchyma sequelae [30] or other underlying co-morbid conditions that affect health for a longer term. We did not perform pulmonary function tests [32], chest radiology, or blood laboratories for investigating the existence of other diseases that may influence the current state of the activity domain. 

There was no evidence of association between the haze/post-haze year and symptom domain. This may be because the symptom domain questions asked about the respiratory symptom experienced in the past, and most of the respondents experienced similar symptoms. Though not significant, respondents starting treatment during the peak of haze (corresponding to PM_10_ index exposure level of 40–55), appeared to have higher symptoms score. This result is in accordance with a study by Muniyandi [8], who found symptoms, such as breathlessness, cough, and chest pain, were still experienced by TB patients one-year after treatment. Moreover, there is no evidence of association between PM_10_ exposure level to impact domain. Compared with other domain scores, this impact domain score was lower. While the disease itself is already cured, patients may still experience psycho-social aspects, such as lack of energy or a feeling that exercise is not safe, but the different levels in personal, cultural, and social support among respondents may have an influence in this domain [34]. 

In our study, males showed higher activity and total domain scores, possibly owing to their higher prevalence of smoking. There is doubt whether sex has an independent role in HRQoL. A meta-analysis by Guo [3] included studies by Muniyandi in India and by Yang in China, indicating that females tend to report poorer health. However, studies by Ting Li [35] in Kiribati and Pasipanodya [30] in the USA have indicated that sex has no role in HRQoL. The sex difference related to disease perception may differ among different cultures. 

In accordance with the results of other studies [3,32,36,37], being of older age and a smoker were significantly associated with worse HRQoL in all domains in our study. There is a possibility of some of the patients developing bronchial and parenchyma sequelae, which occur despite microbiological cure due to aging [30]. Indonesia has the highest smoking rates in the world. The male smoking rate in Indonesia is about 76%, much higher than that of females of 3.6% [38]. Smoke contains particulate matter and other toxic chemical substances that are already known as a risk for lower HRQoL. The smoking behavior in many workplaces or indoor rooms contributes, with a higher amount of secondhand smoke. Moreover, secondhand smoke was associated with lower HRQoL more significantly in women [39]. Thus, an integrated TB treatment and smoking cessation program should be considered to improve HRQoL of TB cases [40]. In addition, providing intensive health promotion among the older and smoker group, such as having a regular medical examination, may provide benefits for detecting the worst respiratory impairments.

Our study described the respiratory morbidity status among the target population group in Pekanbaru, and the findings may not be generalizable to other populations. Limitations of this study include both selection bias and information bias. As the 2015 haze crisis had already abated for three years and many patients treated during that period were difficult to reach, recruiting the subjects was challenging. The subjects who participated may have been more aware of the importance of TB treatment, and this may not represent the true spectrum of quality of life of former TB cases in the population. As we had informed respondents by phone that the purpose of the study was to measure the current health status and asked them to visit the local Puskesmas, their responses may not have been spontaneous. Therefore, SGRQ reflects perceptions at the current date when the question was asked. Since SGRQ is self-assessed, the answer also depends on the respondent’s intention and personality background and how they perceived their health condition. In addition, as we assume all respondents to be exposed to the same daily average of PM_10_ index, the citywide air quality data may not provide an accurate estimate of individual exposure. Despite these limitations, this study contributes to our understanding of possible long-term effects on respiratory morbidity of air pollution due to haze exposure during TB treatment.

## 5. Conclusions

The subsequent respiratory morbidity status of TB cases in the activity domain was poorer among respondents treated during a haze year (2015) in Pekanbaru, but the effect was limited to those with an average PM_10_ index exposure ≥ 55. Moreover, the respiratory morbidity-related quality-of-life of cases who were older and smokers were significantly worse. This raises concern for monitoring and improving the quality of life of TB patients treated during a haze year. Concern to improve the quality of life of patients should be given to TB patients, particularly among the smoker and older groups.

## Figures and Tables

**Table 1:** Demographic, clinical, and environmental characteristics of the study respondents.

Characteristics	Treated on 2015 *N* = 133 (%)	Treated on 2016 *N* = 103 (%)	*p*-Value
**Sex group**			
Female	31 (23.3)	31 (30.1)	0.30
Male	102 (76.7)	72 (69.9)	
**Age group**			0.11
<33 year	46 (34.6)	34 (33.0)	
33–49 year	37 (27.8)	41 (39.8)	
>49 year	50 (37.6)	28 (27.2)	
**Education group**			0.98
Low	71 (53.4)	56 (54.4)	
High	62 (46.6)	47 (45.6)	
**Occupation group**			
Outdoor job	93 (69.9)	77 (74.7)	0.50
Indoor job	40 (30.1)	26 (25.2)	
**Income group**			1
<median	74 (55.6)	57 (55.3)	
≥median	59 (44.4)	46 (44.7)	
**Smoking group**			0.94
Non-smoker	78 (58.6)	59 (57.3)	
Smoker	55 (41,4)	44 (42.7)	
**Initial AFB group**			
Negative	39 (29.3)	34 (33.0)	0.64
Positive	94 (70.7)	69 (67.0)	
**Conversion Month group**			
Month 2nd	130 (97.7)	98 (95.1)	0.46
Month 3rd	3 (2.3)	5 (4.9)	
**Treatment Result group**			0.51
Completed	74 (55.6)	52 (50.5)	
Cured	59 (44.4)	51 (49.5)	
**PM_10_ exposure group**			< 0.01
<40	0	103 (100)	
40–55	67 (51.0)		
≥55	66 (49.0)		

The value indicated absolute number (percentage).

**Table 2:** Distribution of symptom, activity, impact, and total (SGRQ) domain scores for former tuberculosis (TB) patients two–three years after completion of TB treatment in the study population.

Group	Symptom Score	Activity Score	Impact Score	Total Score
Median haze year (2015) group (IQR)	17.9 (12.3, 30.7)	23.7 * (17.2, 30.9)	15.4 (9.5, 22.4)	19.2 (13.0, 25.4)
Median post-haze year (2016) group (IQR)	17.9 (11.1, 26.6)	18.4 * (11.9, 24.8)	14.6 (9.5, 21.4)	17.7 (12.9, 23.9)

The * indicates a significant difference in the median of score between the haze and post- haze group.

**Table 3:** Coefficient of quantile regression with 95% confidence interval to predict the median of SGRQ domain score from the predictor exposure.

Variables	Level	Symptom Domain	Activity Domain	Impact Domain	Total Domain
Sex group	Female	0	0	0	0
	Male	2.72 (−0.56, 8,35)	1.07 (0.25, 10.58) *	2.00 (−0.07, 5.11)	3.28 (0.75, 6.02) *
Age group	<33 year	0	0	0	0
	33–49 year	3.99 (2.68, 7.24) *	1.23 (−0.22, 7.19)	5.41 (3.52, 7.92) *	5.01 (2.04, 6.52) *
	>49 year	14.82 (11.67, 19.16) *	7.52 (6.41, 16.88) *	7.71 (5.51, 9.47) *	9.49 (6.86, 11.08) *
Education group	Low	0	0	0	0
	High	0.00 (−2.96, 4.89)	0.10 (−1.83, 4.81)	−1.57 (−3.91, 1.12)	−0.15 (−2.36, 2.41)
Occupation group	Outdoor job	0	0	0	0
	Indoor job	−1.14 (−4.73, 6.51)	0.02 (−5.8, 2.81)	0.53 (−1.66, 2.87)	0.54 (−3.38,3.54)
Income group	<median	0	0	0	0
	≥median	0.00 (−4.88, 2.95)	−0.26 (−5.04, 3.98)	−0.15 (−2.52, 2.53)	−0.77 (−3.26, 1.64)
Smoking group	Non-smoker	0	0	0	0
	Smoker	7.84 (3.96, 12.35) *	6.04 (5.66, 6.75) *	5.17 (1.66, 7.85) *	5.43 (1.19, 8.17) *
Initial AFB group	Negative	0	0	0	0
	Positive	−1.07 (−5.77, 2.91)	0.10 (−5.48, 1.79)	0.42 (−2.90, 3.89)	−0.25 (−3.08, 3.08)
Conversion month group	Month 2nd	0	0	0	0
	Month 3rd	−4.88 (−9.88, 17.59)	0.42 (−2.91, 12.53)	0.62 (−2.23, 17.95)	0.58 (−3.81, 17.81)
Treatment result group	Completed	0	0	0	0
	Cured	−2.06 (−6.03, 0.33)	−0.77 (−5.42, 0.51)	0.97 (−1.90, 3.05)	0.31 (−1.96, 2.75)
PM_10_ exposure group	<40	0	0	0	0
	40–55	3.65 (−2.37, 7.87)	0.26 (−0..44, 6.38)	0.51 (−2.18, 3.97)	1.72 (−0.25, 4.57)
	≥55	−0.45 (−2.66, 3.48)	5.67 (0.99, 6.18) *	1.18 (−1.92, 3.64)	−0.13 (−2.69, 4.37)
Year of Treatment	2016	0	0	0	0
	2015	0.00 (−2.33, 6.01)	5.32 (0.12, 6.16) *	0.80 (−2.04, 2.85)	1.49 (−1.01, 4.19)

Values in brackets denote 95% confidence interval. The * indicates a significant difference.

## References

[1] World Health Organization (2017). Global Tuberculosis Report 2017.

[2] Aggarwal A. (2010). Health-related quality of life: A neglected aspect of pulmonary tuberculosis. Lung India.

[3] Guo N., Marra F., Marra C.A. (2009). Measuring health-related quality of life in tuberculosis: A systematic review. Health Qual. Life Outcomes.

[4] Pratiwi P.D., Perwitasari D.A. (2017). Validation of St. George’s Respiratory Questionnaire (SGRQ) in Chronic Obstructive Pulmonary Disease (COPD) at Respira Lung Hospital Yogyakarta. J. Manag. Pharm. Pract..

[5] Wahyuni A.S., Soeroso N., Harahap J., Amelia R., Alona I. (2018). Quality of life of pulmonary TB patients after intensive phase treatmentin the health centers of Medan city, Indonesia. IOP Conf. Ser. Earth Environ. Sci..

[6] Mamani M., Majzoobi M.M., Ghahfarokhi S.M., Esna-Ashari F., Keramat F. (2014). Assessment of health-related quality of life among patients with Tuberculosis in Hamadan, Western Iran. Oman Med. J..

[7] Hama M., Ushiki A., Kosaka M., Yamazaki Y., Yasuo M., Yamamoto H., Hanaoka M. (2016). Health-related quality of life in patients with pulmonary non-tuberculous mycobacteria infection. Int. J. Tuberc. Lung Dis..

[8] Muniyandi M., Rajeswari R., Balasubramanian R., Nirupa C., Gopi P.G., Jaggarajamma K., Sheela F., Narayanan P.R. (2007). Evaluation of post-treatment health-related quality of life (HRQoL) among tuberculosis patients. Int. J. Tuberc. Lung Dis..

[9] Ferrer M., Villasante C., Alonso J., Sobradillo V., Gabriel R., Vilagut G., Masa J.F., Viejo J.L., Jiménez-Ruiz C.A., Miravitlles M. (2002). Interpretation of quality of life scores from the St George’s Respiratory Questionnaire. Eur. Respir. J..

[10] Sherpa C.T., LeClerq S.L., Singh S., Naithani N., Pangeni R., Karki A., Chokhani R., Han M., Gyetko M., Tielsch J.M. (2015). Validation of the St. George’s Respiratory Questionnaire in Nepal. Chronic Obstr. Pulm. Dis. J. COPD Found..

[11] Guillerm N., Cesari G. (2015). Fighting ambient air pollution and its impact on health: From human rights to the right to a clean environment. Int. J. Tuberc. Lung Dis..

[12] Pinichka C., Makka N., Sukkumnoed D., Chariyalertsak S., Inchai P., Bundhamcharoen K. (2017). Burden of disease attributed to ambient air pollution in Thailand: A GIS-based approach. PLoS ONE.

[13] WHO Ambient (Outdoor) Air Quality and Health. http://www.who.int/en/news-room/fact-sheets/detail/ambient-(outdoor)-air-quality-and-health.

[14] Kusumaningtyas S.D.A., Aldrian E. (2016). Impact of the June 2013 Riau province Sumatera smoke haze event on regional air pollution. Environ. Res. Lett..

[15] Koplitz S.N., Mickley L.J., Marlier M.E., Buonocore J.J., Kim P.S., Liu T., Sulprizio M.P., DeFries R.S., Jacob D.J., Schwartz J. (2016). Public health impacts of the severe haze in Equatorial Asia in September–October 2015: Demonstration of a new framework for informing fire management strategies to reduce downwind smoke exposure. Environ. Res. Lett..

[16] Yang W., Yuan G., Han J. (2019). Is China’s air pollution control policy effective? Evidence from Yangtze River Delta cities. J. Clean. Prod..

[17] Indonesia Decree of the Head of the Environmental Impact Control No. 107/1997 on Calculation, Reporting and Information of Air Quality Index. http://www.cets-uii.org/BML/Udara/ISPU/ISPU%20(Indeks%20Standar%20Pencemar%20Udara).htm.

[18] Greenstone M. Indonesia’s Worsening Air Quality and Its Impact on Life Expectancy. https://aqli.epic.uchicago.edu/wp-content/uploads/2019/03/Indonesia-Report.pdf.

[19] Popovic I., Soares Magalhaes R.J., Ge E., Marks G.B., Dong G.H., Wei X., Knibbs L.D. (2019). A systematic literature review and critical appraisal of epidemiological studies on outdoor air pollution and tuberculosis outcomes. Environ. Res..

[20] Smith G.S., Schoenbach V.J., Richardson D.B., Gammon M.D. (2014). Particulate air pollution and susceptibility to the development of pulmonary tuberculosis disease in North Carolina: An ecological study. Int. J. Environ. Health Res..

[21] Zhu S., Xia L., Wu J., Chen S., Chen F., Zeng F., Chen X., Chen C., Xia Y., Zhao X. (2018). Ambient air pollutants are associated with newly diagnosed tuberculosis: A time-series study in Chengdu, China. Sci. Total Environ..

[22] Chuang H.C., Chen K.Y., Chuang K.J., Liu H.C., Lee K.Y., Feng P.H., Su C.L., Lin C.L., Lee C.N. (2016). Particulate matter is associated with sputum culture conversion in patients with culture-positive tuberculosis. Ther. Clin. Risk Manag..

[23] Jung J.H., Kang I.G., Cha H.E., Choe S.H., Kim S.T. (2012). Effect of Asian Sand Dust on Mucin Production in NCI-H292 Cells and Allergic Murine Model. Otolaryngol. Head Neck Surg..

[24] Maestrelli P., Canova C., Scapellato M., Visentin A., Tessari R., Bartolucci G., Simonato L., Lotti M. (2011). Personal exposure to particulate matter is associated with worse health perception in Adult Asthma. J. Investig. Allergol. Clin. Immunol..

[25] Indonesian Haze: Why It’s Everyone’s Problem—CNN.com. http://edition.cnn.com/2015/09/17/asia/indonesian-haze-southeast-asia-pollution/index.html.

[26] Welling J.B.A., Hartman J.E., Ten Hacken N.H.T., Klooster K., Slebos D.J. (2015). The minimal important difference for the St George’s Respiratory Questionnaire in patients with severe COPD. Eur. Respir. J..

[27] Jones P.W., Forde Y. St George’s Respiratory Questionare Manual. http://www.healthstatus.sgul.ac.uk/SGRQ_download/SGRQ%20Manual%20June%202009.pdf.

[28] Agustine I., Yulinawati H., Gunawan D., Suswantoro E. (2018). Potential impact of particulate matter less than 10 micron (PM_10_) to ambient air quality of Jakarta and Palembang. IOP Conf. Ser. Earth Environ. Sci..

[29] Textor J., van der Zander B., Gilthorpe M.S., Liśkiewicz M., Ellison G.T.H. (2016). Robust causal inference using directed acyclic graphs: The R package ‘dagitty’. Int. J. Epidemiol..

[30] Pasipanodya J.G., Miller T.L., Vecino M., Munguia G., Bae S., Drewyer G., Weis S.E. (2007). Using the St. George Respiratory Questionnaire to ascertain health quality in Persons with treated pulmonary tuberculosis. Chest.

[31] Perwitasari D.A., Mulyani U.A., Thobari J.A. Pengukuran Kualitas Hidup Pasien Tuberculosis Menggunakan Instrumen St Geotge Respiratory Questionare (SGRQ) di Yogyakarta. http://eprints.uad.ac.id/6621/.

[32] Rekha V.V.B., Ramachandran R., Rao K.V.K., Rahman F., Adhilakshmi A.R., Murugesan P., Sundaram V., Narayanan P.R. (2009). Assesment of long term status of sputum positive pulmonary TB patients succesfully treated with short course chemotherapy. Indian J. Tuberc..

[33] Obaseki D., Erhabor G., Awopeju O., Obaseki J., Adewole O. (2013). Determinants of health related quality of life in a sample of patients with chronic obstructive pulmonary disease in Nigeria using the St. George’s respiratory questionnaire. Afr. Health Sci..

[34] de Farias S.N.P., da Silva Medeiros C.R., Paz E.P.A., de Lobo A.J.S., Ghelman L.G. (2013). Completeness in caring: Study of quality of life in clients with tuberculosis. Esc. Anna Nery.

[35] Li C.T., Chu K.H., Reiher B., Kienene T., Chien L.Y. (2017). Evaluation of health-related quality of life in patients with tuberculosis who completed treatment in Kiribati. J. Int. Med. Res..

[36] Brown J., Capocci S., Smith C., Morris S., Abubakar I., Lipman M. (2015). Health status and quality of life in tuberculosis. Int. J. Infect. Dis..

[37] Campos A.C.V., Ferreira E.F.E., Vargas A.M.D., Albala C. (2014). Aging, Gender and Quality of Life (AGEQOL) study: Factors associated with good quality of life in older Brazilian community-dwelling adults. Health Qual. Life Outcomes.

[38] Smoking Rates by Country Population 2019. http://worldpopulationreview.com/countries/smoking-rates-by-country/.

[39] Bridevaux P.O. (2007). Secondhand smoke and health-related quality of life in never smokers: Results from the SAPALDIA Cohort Study 2. Arch. Intern. Med..

[40] Awaisu A., Haniki Nik Mohamed M., Noordin N., Muttalif A., Aziz N., Syed Sulaiman S., Mahayiddin A. (2012). Impact of connecting tuberculosis directly observed therapy short-course with smoking cessation on health-related quality of life. Tob. Induc. Dis..

